# Delay in Adélie penguin nest occupation restricts parental investment in nest construction and reduces reproductive output

**DOI:** 10.1002/ece3.10988

**Published:** 2024-03-11

**Authors:** Madi J. McLatchie, Louise Emmerson, Simon Wotherspoon, Colin Southwell

**Affiliations:** ^1^ Department of Climate Change, Energy, the Environment and Water Australian Antarctic Division Kingston Tasmania Australia; ^2^ Institute for Marine and Antarctic Studies University of Tasmania Hobart Tasmania Australia

**Keywords:** nest structure, nest survival, remotely operated cameras, reproductive success, seabird ecology, Windmill Islands

## Abstract

Reproductive success is an important demographic parameter that can be driven by environmental and behavioural factors operating on various spatio‐temporal scales. As seabirds breed on land and forage in the ocean, processes occurring in both environments can influence their reproductive success. At various locations around East Antarctica, Adélie penguins' (*Pygoscelis adeliae*) reproductive success has been negatively linked to extensive sea‐ice. In contrast, our study site in the Windmill Islands has limited fast ice present during the breeding season, allowing us to examine drivers of reproductive success under vastly different marine environmental conditions. Here, we examined the reproductive success of 450 Adélie penguin nests over a 10‐year period using images obtained from remotely operated cameras. We analysed nest survival in relation to marine and climatic factors, environmental conditions at the camera site and immediately around the nest, and behavioural attributes reflecting parental investment and phenological timing. Our key result was a strong positive association between nest structure and chick survival, particularly when ground moisture and snow cover around the nest were high. Earlier nesting birds were more likely to build bigger nests, although it is unclear whether this is due to more time available to build nests or whether early arrival and high‐quality nests are complementary traits. This intrinsic activity is likely to become more important if future predictions of increased snowfall in this region manifest.

## Graphical abstract

In the Windmill Islands, it is not well‐known what drives Adélie penguin reproductive success. Here, we utilised remotely operated cameras to determine the drivers of this and found that adult behaviour largely influences nest survival and reproductive success.

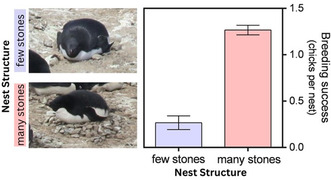

## 1 INTRODUCTION

Globally, seabirds are highly studied marine organisms that are well‐known as bioindicators of ecosystem change (Croxall et al., 2012) in their terrestrial breeding habitats and marine foraging grounds (Sydeman et al., 2012). Seabirds are sensitive to natural and anthropogenic factors influencing their habitats and populations, ranging from reductions in prey availability through fisheries activities (Cairns, 1988; Hátún et al., 2017) and localised prey depletions (Sherley et al., 2018) to ingestion of plastics and pollutants (Jepson et al., 2016; Kühn et al., 2015) and reduction of breeding habitat quality due to storms (de Villiers, 2002). Terrestrial and marine factors directly or indirectly affect seabird survival, recruitment and fecundity, and through these, population growth (Barbraud et al., 2018; Richards et al., 2021; Southwell et al., 2021). Because overall population change integrates key demographic parameters including reproductive success, understanding the short‐term response of demographic parameters is a critical element in developing an understanding of the overall drivers of population change (Fayet et al., 2021; Labrousse et al., 2021). Reproductive success may be driven by both extrinsic (environmental) and intrinsic (behavioural) factors (Glencross et al., 2021; Morandini et al., 2021) operating on various spatial and temporal scales (Barreau et al., 2019; Caravaggi et al., 2019; Emmerson & Southwell, 2022).

**FIGURE 1:**
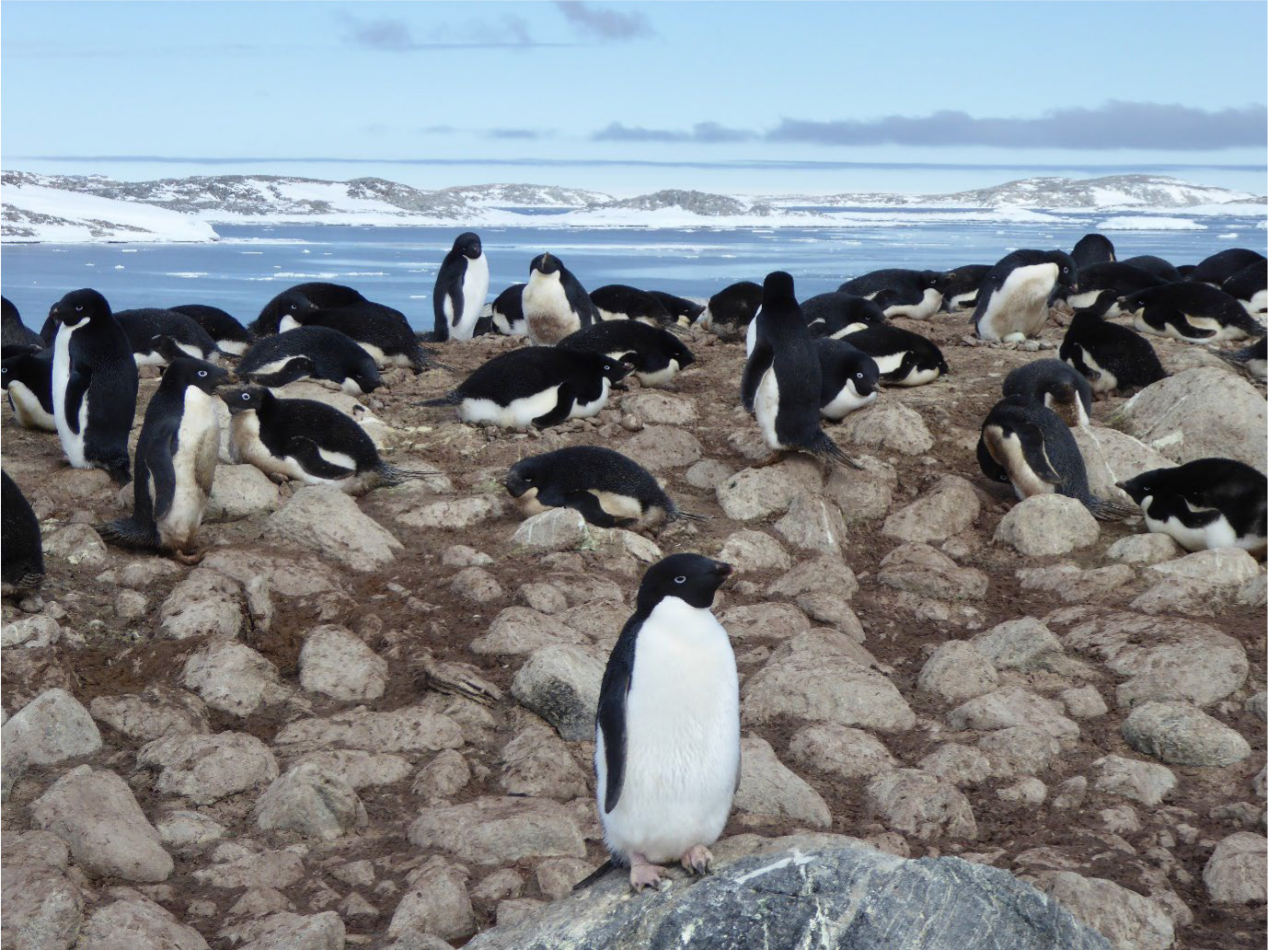
Adélie penguin breeding colony at Odbert Island, Windmill Islands.

The Southern Ocean is a unique and highly productive environment that supports a diverse range of seabird populations (Constable et al., 2014). It is also an area undergoing rapid environmental shifts that can impact the higher trophic levels including seabirds (Constable et al., 2014; Murphy et al., 2007). Most seabird species inhabiting these ecosystems are adapted to breed during the relatively short austral summer when environmental conditions are more conducive and have developed behaviours to allow them to successfully reproduce (Smetacek & Nicol, 2005). The Adélie penguin *Pygoscelis adeliae* (Figure 1), the focal species of this study, is an extensively studied pagophilic marine predator with a circumpolar breeding distribution (Ainley, 2002), and is a key indicator of marine ecosystem status monitored by the Convention for the Conservation of Antarctic Marine Living Resources (CCAMLR) Ecosystem Monitoring Program (CEMP) (Agnew, 1997). Breeding on areas of ice‐free land along the Antarctic coastline, they exhibit high site philopatry (Ainley, 2002). During the austral spring, they build nests out of stones gathered during the courtship period (Tenaza, 1971), and in East Antarctica, the region of interest here, are central‐place foragers feeding predominantly on krill and fish (Clarke et al., 2006; Kokubun et al., 2021; Wienecke et al., 2000).

Previous studies have found that Adélie penguin reproductive success can be influenced by external factors operating in their marine and terrestrial environments. For example, reproductive success can be negatively impacted by the presence of extensive fast ice adjacent to their colonies which forces the penguins to walk across ice to their foraging grounds, requiring more time and energy, in numerous sites including Pointe Géologie (Barreau et al., 2019), Béchervaise Island (Emmerson & Southwell, 2008), Lutzow‐Holm Bay (Kato et al., 2002) and at Ross Island in the Ross Sea (Ninnes et al., 2011). Reduced pack ice can also influence reproductive success through reduced foraging success, resulting from less under‐ice microalgal communities that sustain krill populations (Barreau et al., 2019). At their nesting sites, snowfall and moisture can impact Adélie penguins' ability to successfully breed if heavy snow cover prevents access to nest sites when adults arrive at the beginning of the breeding season (Ainley et al., 2010; Lynch et al., 2009), or through the wetting of young chick's bodies leading to reduced survival (Chapman et al., 2011). As well as reproductive success, penguin phenology has been influenced by the nesting habitat in colonies, including on Torgersen and Humble Islands where island geomorphology caused higher snow accumulation leading to delayed clutch initiation at Torgersen (Cimino et al., 2019). The magnitude and effect of these factors likely vary spatially depending on the distance between the breeding site and foraging grounds, and also the marine resources available for individuals during the breeding season.

Although adverse environmental conditions such as those described above may affect Adélie penguin's reproductive success, the way parents respond behaviourally can play a major role in ameliorating against these, as well as enabling them to adapt to environmental change. Phenological timing is known to effect reproductive success, where late breeding is disadvantageous (Smiley & Emmerson, 2016). Furthermore, decisions by the parents on their investment in building and maintaining their nests, choice of nest location (Morandini et al., 2021), as well as their presence at the nest and the ability to coordinate between partners (Davis, 1988) are therefore likely to matter for chick survival. In colonial species, more experienced or older breeding penguins often dominate the highest quality nesting sites, leaving poorer quality sites to subordinate individuals (the ideal despotic model: Fretwell & Lucas, 1969) and thereby ultimately maximising fitness in a spatially heterogenous habitat (Oro, 2008). In *Pygoscelid* penguins, the more experienced or older birds select nests further into the colony away from the periphery (Spurr, 1975), and these central nests are typically larger with more nest stones, expend less energy defending their nests from pebble‐stealing (Morandini et al., 2021), and have a higher breeding success compared to their peripheral‐nesting counterparts (Morandini et al., 2021; Penney, 1968; Tenaza, 1971). Nesting further into the colony at Capes Crozier and Royds had a more positive impact on reproductive success compared to colony size and geomorphological variables like aspects (Schmidt et al., 2021). Parental coordination is particularly important for new hatchlings and small chicks as they are ectothermic which makes them particularly vulnerable to harsh environmental conditions, are susceptible to skua predation, and require frequent small meals (Ainley, 2002; Olmastroni & Pezzo, 2004; Smiley & Emmerson, 2016). At this stage, chick survival may be enhanced if adults invest in protecting the young chicks from weather and predators (Ainley, 2002). Skua predation can reduce egg and chick survival (Young & Millar, 1999), and nest failure can occur through egg loss during incubation due to storms displacing eggs from the nest via snowmelt runoff or eggs being crushed during adult disputes (Davis, 1988). Nest maintenance, location and parental coordination are all critical components of the chick's success, which becomes less important post‐guard when chicks can be left unattended and gather in tight groups known as crèches (Young & Millar, 1999) for thermoregulation and protection against skua predation (Lawless et al., 2001).

Overall breeding success variability between years and across sites results from multiple forces acting upon the birds' breeding site and foraging grounds. In this study, we assess reproductive success for Adélie penguins over multiple consecutive years across a large regional population in East Antarctica to identify these forces and understand the role of environmental and behavioural factors. Specifically, we assess their reproductive success across the Windmill Islands, where a network of automated cameras has been established (Newbery & Southwell, 2009; Southwell & Emmerson, 2015) to obtain daily records of nest activity. This regional population, currently numbering around 190,000 breeding pairs, has increased substantially over the past six decades with a recent slowing likely due to breeding habitat limitations or food availability through intra‐specific competition (Southwell et al., 2021). In contrast to other East Antarctic locations where fast ice is extensive and is a primary driver of reproductive success (Barreau et al., 2019; Emmerson & Southwell, 2008; Ninnes et al., 2011), the Windmill Islands has relatively little fast ice present next to their colonies during the penguin breeding season. Therefore, we expect that reproductive success in this region will be driven by environmental and behavioural factors other than fast ice, although it is not clear whether these factors are marine and climatic, terrestrial or behavioural, and under what conditions reproductive success increases with greater parental investment. Here, we assess nest camera images to determine reproductive success and nest conditions or attributes that likely reflect parental investment at 450 Adélie penguin nests, located at five camera sites, across a 10‐year period. The objectives of the study were to (1) assess the spatio‐temporal variability of reproductive success across the Windmill Islands group; and (2) identify the drivers of nest survival in terms of marine and climatic conditions, terrestrial conditions and behavioural attributes.

## 2 MATERIALS AND METHODS

### 2.1 Study region

The Windmill Islands lie alongside a north–south oriented 35 km coast off Wilkes Land, East Antarctica (66°15′ S, 110°33′ E). The island group is made up of 272 islands and five continental peninsulas, the largest of which is 396 ha and the furthest is 18 km from the coast. The islands and peninsulas consist of low (maximum elevations of around 90 m a.s.l.) rounded hills and intervening valleys filled with snow or glacial moraine and exfoliated detritus (Blight & Oliver, 1977). The region has a large area of ice‐and snow‐free land (Fraser et al., 2012) and easy beach access, providing a suitable breeding habitat for Adélie penguins. Approximately 5% of Antarctica's population of Adélie penguins breed in the Windmill Islands across 15 islands (Southwell et al., 2021). Surveys of the region in the 1960s (Orton, 1963), 1980s/1990s (Woehler et al., 1991) and 2010s (Southwell & Emmerson, 2020) have shown that the population has increased by a factor of six in the last 60 years (Southwell & Emmerson, 2020). These populations are separated from other large East Antarctic populations by glaciers for several 100 km to the east and west (Southwell et al., 2021).

### 2.2 Study species

The Adélie penguin has a well‐known and predictable breeding season where birds arrive in mid‐late October, with chicks hatching in late December, and the adults and fledglings departing from mid‐late February and early March (Ainley, 2002; Emmerson et al., 2011). Therefore, for this study, we considered three distinct phases of the breeding cycle: incubation (15 November to 31 December), guard (1 January to 15 January) and crèche (January 15 onwards).

### 2.3 Remotely operating cameras and nest selection

**FIGURE 2:**
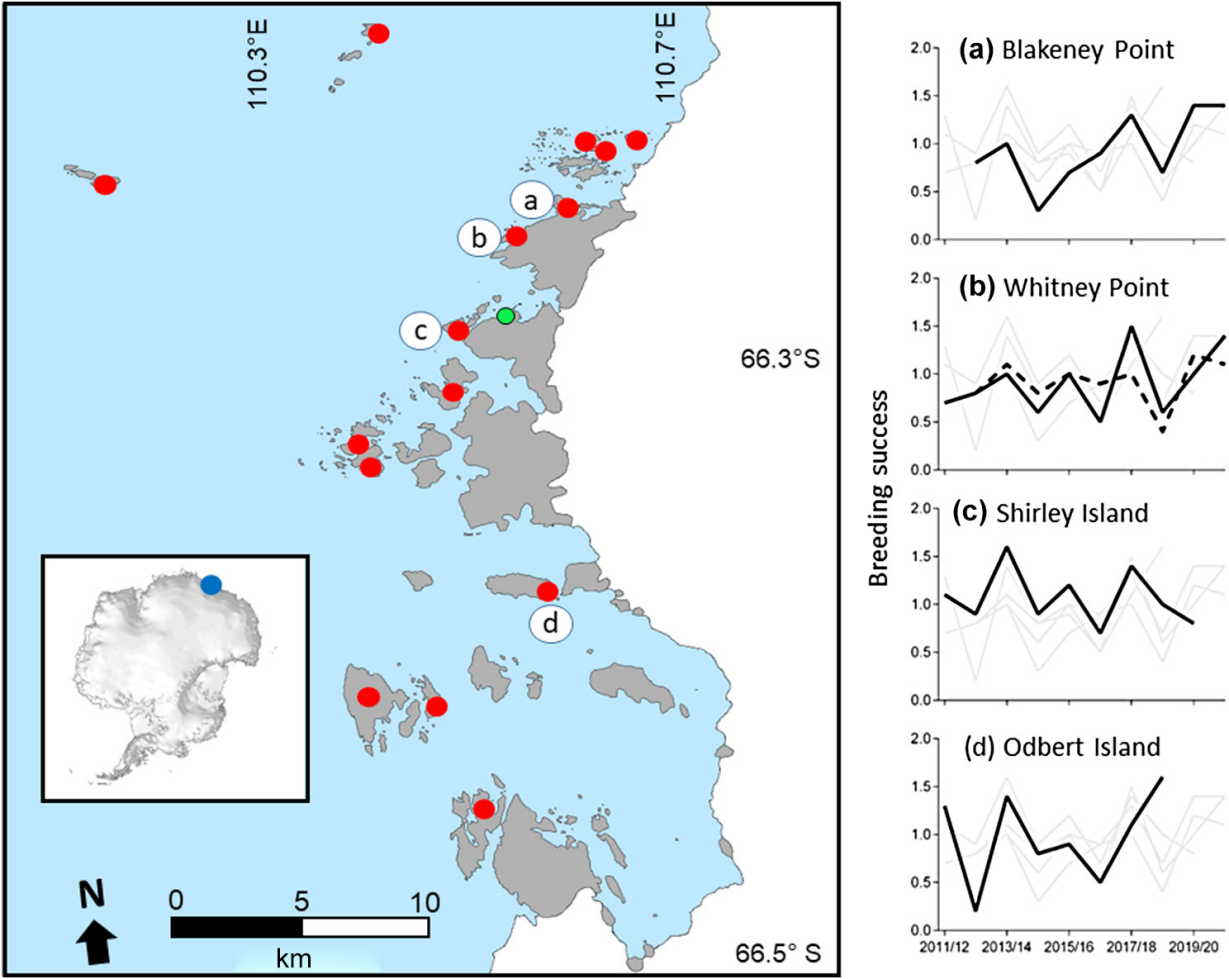
Map of the Windmill Islands study region (main panel) and its location in Antarctica (inset), with breeding success (chicks crèched per nest) across camera sites and seasons (panels a–d). In the main panel, the green dot indicates Casey Station, red dots indicate all breeding sites, lettered circles indicate four breeding sites where five cameras are located, grey shading represents ice‐free land, white shading is continental ice and blue shading is ocean. In panels a–d, each panel shows data for all cameras where data for the specified site are shown in black and other sites in grey. Blakeney Point, Shirley Island and Odbert Island have one camera each; Whitney Point has two cameras. In panel b, Whitney Point camera 1 is the solid line and Whitney Point camera 2 is the dashed line.

Five remotely operated cameras were established at four breeding sites in the Windmill Islands region in 2011/12 to monitor Adélie penguin colonies as part of a broader network across East Antarctica (Southwell & Emmerson, 2015). The camera sites included one camera at Blakeney Point, Shirley Island and Odbert Island and two camera sites at Whitney Point (Figure 2). Cameras were located 15–30 m from the edge of breeding colonies and the fields of view included a minimum of 30 nests. We hereafter use the term ‘camera site’ to refer to the field of view of a camera rather than the specific location of a camera, and the term ‘breeding site’ to refer to an entire location of ice‐free land where a breeding population occurs. In general, camera sites were located at mid‐level elevations (20–40 m a.s.l.), had gentle slopes with aspects ranging from north‐west to south, and had micro‐habitats of small boulders and stones embedded in or lying on guano‐covered ground. None of the camera sites were located on prominent ridges or gullies. The Blakeney Point and Shirley Island camera sites had higher elevations than other camera sites, and the Blakeney Point and Odbert Island camera sites had slightly steeper slopes.

Each camera consists of a Canon™ EOS‐300D or 1000D digital single lens reflex camera with Pelican™ weather‐proof housing, a window with an external cover to protect against abrasions, a programmable controller, a surveyor's tripod with elevation control and a plastic mat to restrain the tripod to the ground using rocks as described in Southwell and Emmerson (2015). The cameras were programmed to take 10 photos per day over the breeding season (October to March), with photos being taken every hour for a 10‐hour period centred around solar midday. In this study, we analysed images from the 10 breeding seasons between 2011/12 and 2020/21, though data for some cameras in certain years were unavailable due to camera operation failures (Appendices B). The images were processed using SPPY‐CAMS software (V 1.0.0) (Cusick et al., 2019).

Observations were made for 10 randomly selected active nests for each camera site and season combination. We randomly selected nests each season to maximise the variation in nest‐specific environmental conditions in the observed sample of nests. Although individuals are known to show nest fidelity between years, we did not account for this due to slight differences in nest locations each season and being unable to confirm whether the same individual is at a nest year after year. A nest was considered active if it had a penguin present 3 days before, on, and after 1 December. Nests were selected using an x‐y axis and a random number generator of x and y coordinates to identify study nests in the camera field of view. The selected nests were used to assess nest‐specific measures or characteristics and to record reproductive success (described below).

### 2.4 Reproductive success

We used two metrics of reproductive success which we refer to as breeding success and nest survival to crèche. Breeding success for each nest was recorded as either 0, 1 or 2 chicks reaching crèche, where 0 indicates nest failure. Nest survival was measured as binomial data (0,1) where 0 is a fail and 1 indicates at least one chick reaching the crèche stage in the nest. Nest failure occurs when either the parents abandoned the nest, both eggs failed to hatch, or the chicks died. Crèching was differentiated from nest failure by identifying crèches in the nest vicinity and assessing whether the chick was of crèching size prior to it no longer being observed at the nest. If a chick was not of crèche size at the time it was no longer observed at the nest, it was considered to have failed. The date of success (i.e., crèche date) or failure was recorded for each nest.

### 2.5 Environmental covariates

We considered a total of nine environmental covariates describing key environmental processes as candidate drivers of reproductive success operating at a range of spatial scales (regional, camera site, nest).

Marine and climatic covariates included the Southern Oscillation Index (SOI), Southern Annular Mode (SAM), fast ice extent and the potential for overlap in penguin foraging ranges with neighbouring colonies. The SOI and SAM are climate indices of the Southern Ocean and Antarctic climate system derived from atmospheric pressure measurements (Rogers et al., 2020), and were included due to their influence on Southern Ocean weather patterns and previous studies finding it to be a significant driver for phenology (Emmerson et al., 2011; Hindell et al., 2012) and breeding success (Descamps et al., 2016; La Cock, 1986; Price et al., 2020). Positive SOI and SAM values are both characterised by cool conditions across East Antarctica (Kwok & Comiso, 2002; Thompson et al., 2011). SOI and SAM values were averaged for two periods: October to January to correspond with the Adélie penguin reproductive cycle, and July to June for a winter value prior to the breeding season. Data were obtained for SAM from the NOAA Climate Prediction Centre (https://www.cpc.ncep.noaa.gov/products/precip/CWlink/daily_ao_index/aao/monthly.aao.index.b79.current.ascii.table), and SOI from the Australian Bureau of Meteorology (BOM) (http://www.bom.gov.au/climate/enso/soi/).

Fast ice forms around local topographic and bathymetric features (Fraser et al., 2012) and the variation this causes in the extent of fast ice could differentially influence reproductive success at the camera sites included in this study. We obtained shapefiles of the fast‐ice distribution in the Windmill Islands from the Natice website (www.natice.noaa.gov) and measured the shortest distance between the four breeding sites and the fast‐ice edge in ArcGIS during mid‐November for each breeding season of the study timeframe (2011/12 to 2020/21).

Because of the potential for overlapping foraging ranges between penguins breeding at nearby colonies to result in intra‐specific competition, we included a covariate of ‘potential foraging overlap’ based on values presented in Southwell et al. (2021) for each breeding site. Values were estimated using the ‘foraging radius approach’ developed by Critchley et al. (2018) and Handley et al. (2021) and based on recent population estimates for the region's breeding sites, the distance between penguin breeding sites and foraging distances from colonies to estimate the degree of potential overlap between local breeding penguins when foraging at sea.

Terrestrial covariates included windchill, and ground moisture and snow cover at individual nests. To observe the effects of windchill (°C) on Adélie breeding success, we calculated the incubation, guard and crèche mean windchill each breeding season from 2011/12 to 2020/21. Windchill temperatures were calculated from ambient air temperature, wind speed and relative humidity recorded at Casey Station (Figure 2) using the formula provided by the Australian Bureau of Meteorology (BoM) (http://www.bom.gov.au/info/thermal_stress/).

**FIGURE 3:**
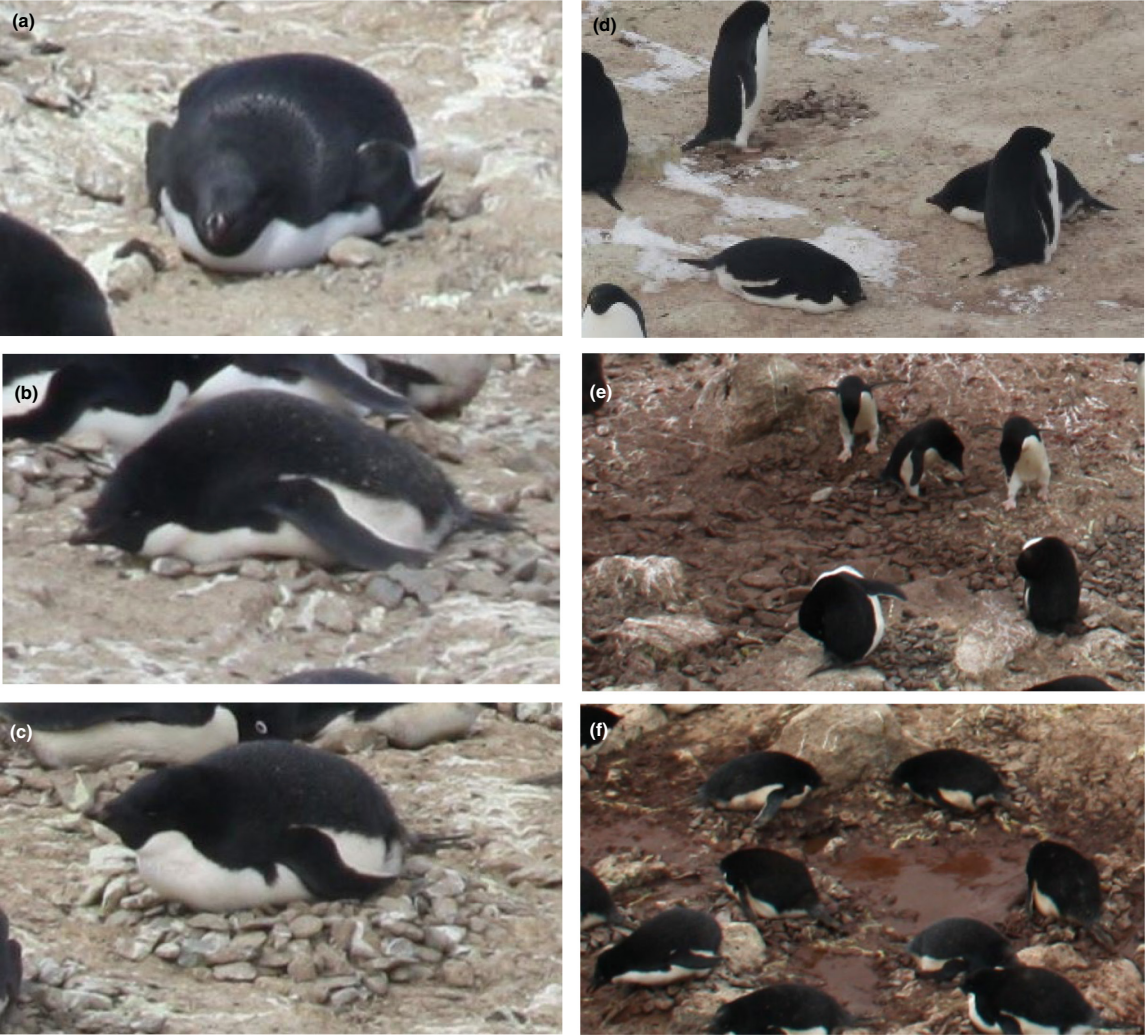
Nest structure and ground moisture scores examples. (a) Nest structure score of 1; (b) Nest structure score of 2; (c) Nest structure score of 3; (d) No ground moisture; (e) Low ground moisture; (f) High ground moisture.

Snow cover and ground moisture were recorded every day for individual nests from the nest occupation date (NOD, see below) to the nest fail or chick crèche date. These were recorded daily in categories of 0–3, where 0 is no snow cover or dry ground, 1 is a light snow cover or low moisture, 2 is a moderate snow cover or moderate moisture and 3 is a heavy snow cover or high moisture (Figure 3). These were assessed from both within the immediate ground surrounding the nest (~30 cm) and in the nest itself. From these records, the number of snow and ground moisture days for each category during incubation, guard and crèche were determined for each nest and the nest's propensity to have snow cover or be wet was calculated as the proportion of days during the observation period when each nest had snow or was moist. This allowed us to observe a nest's propensity to experience snow cumulatively over the breeding season, while daily observations showed what nests experienced in relation to its success or failure time.

### 2.6 Behavioural covariates

Finally, we recorded four behavioural variables and attributes for each study nest from the daily camera images. These covariates included nest occupation date (NOD), date of female departure after laying eggs (referred to as the FFT: first foraging trip), nest structure and nest location.

The NOD and FFT date were recorded for each nest. The NOD was recorded as the first day of a continuous period of 5 days with a penguin present at the nest. This was done to reduce the risk of including non‐breeders who have reoccupied the colony and are building ‘practice nests’ (McInnes et al., 2016). The FFT date was identified as the date when the female left the nest for more than five consecutive days, leaving the male to incubate the eggs and was recorded as the day the female was first absent from the nest.

The structure of a nest may provide protection for the chick against detrimental weather conditions like moisture or snow cover (Fargallo et al., 2001), while the location of an individual nest within a colony may influence skua predation or level of disturbance from other penguins (Tenaza, 1971). We recorded nest structure and nest location for each nest on three dates: 1 December, 1 January and 15 January (incubation, guard and crèche periods respectively). Nest structure was assessed on whether stones or pebbles were stacked to create a nest bowl with a score of 1, 2 or 3 (Figure 3) where 1 represents a nest with little or no stones, 2 represents a nest with some stones but not stacked off the ground and 3 represents a nest where multiple stones are stacked with the nest bowl off the ground. Nest location was measured as the number of active nests from the periphery of the colony (1 = edge of the colony, and the location number increasing towards the colony centre as a continuous variable).

### 2.7 Statistical analysis

We used a 3‐step analytical approach to (1) assess spatio‐temporal variation in breeding success between breeding seasons and camera sites, (2) determine whether our marine and climatic, terrestrial, and behavioural covariates were related to nest survival, (3) further explore the relationships and processes underlying the covariates found to be associated with nest survival.

To assess the spatio‐temporal variation in breeding success, we fitted multinomial Log‐linear models using an analysis of deviance to breeding success data. Here, a multinomial metric for breeding success was used as the response variable and camera site, breeding season and their interaction as explanatory variables.

To investigate the drivers of nest survival in relation to our suite of covariates given the spatio‐temporal variation found in the above analysis, we applied Cox proportional hazards models (Cox PH models) (Klein & Moeschberger, 2003) to examine the influence of all environmental and behavioural covariates on nest survival within the breeding season. To do so, we first constructed three sub‐models which included covariates for marine and climatic (SOI, SAM, fast ice extent, potential foraging overlap), terrestrial (windchill, number of ground moisture or snow cover days and propensity for ground moisture and snow cover at individual nests) and behaviour (NOD, FFT, nest structure, nest location). We then constructed a commensurate model using the covariates that were identified as significant in each of the three sub‐models as our final model.

The commensurate Cox PH model allowed understanding at a finer temporal resolution (daily) within the breeding season using binomial data. In this survival analysis, models were fitted to the time of nest failure rather than the time of chick mortality because the date of overall nest failure could be reliably determined from the images, whereas the exact date of individual chick mortality could not and nest failure could also occur during the incubation stage when eggs were present. Nests with at least one surviving chick at the end of the crèche period were considered to have survived and were censored (they were not observed to fail within the study period).

Survival models relate the probability $S\left(t\right)$ an individual nest ‘survives’ until time *t* to the hazard h(*t*), the instantaneous risk of mortality, through the following equation:

$$S\left(t\right)=\exp\left(-\int^{t}h\left(\tau \right)\mathrm{d\tau}\;\right).$$

Here, the hazard represents the instantaneous risk of nest failure, the integral of the hazard represents the cumulative risk experienced up until the current time, and the survival declines exponentially with the cumulative risk of nest failure (Kleinbaum & Klein, 1996). Therefore, an increased hazard at any time prior to time *t* results in a reduced probability of subsequent survival through until time *t*. The Cox PH model assumes the hazard h(*t*) may be represented as the product of a baseline hazard *h*_0_(*t*) denoting a common hazard experienced by all nests (the instantaneous mortality rate with no covariate effects), and a multiplicative factor exp(*Xβ*) that scales the baseline hazard to reflect the effect of the covariates.

$$ℎ({\text{34𝑡)=ℎ}}_{0}\text{(34𝑡𝑡)exp(34𝑡𝑡𝑋34𝑡𝑡𝑋𝛽),}$$

where *X* is a design matrix constructed from the model covariates and *β* is a vector of model coefficients. The proportional hazards assumption implies the effect of the covariates on the hazard is consistent over time. This assumption can be tested by examining the smoothed model residuals over time (Grambsch & Therneau, 1994). To allow for minor departures from the proportional hazards assumption, the three breeding stages (incubation, guard and crèche) were treated as separate strata, and separate model coefficients for selected explanatory covariates were fitted for each stratum. This allows the impact of selected covariates to vary between breeding stages. The model output is a hazard ratio (the measure of the effect of the covariate on nest survival over time) and can be interpreted as: <1 = reduction in hazard, increase in survival; >1 = increase in hazard, decrease in survival; = 1 = no effect. Significant terms are *p* < .05.

Finally, we performed additional analyses including analysis of deviance, ANOVA and linear regression to assess the relationships between important covariates to further understand their role in driving reproductive success and inter‐annual and between‐site differences. Simple one‐way ANOVAs were performed to assess variation amongst years and camera sites for continuous covariate data (e.g., nest location), while for count data (e.g., number of ground moisture days) a (quasi) Poisson GLM with an analysis of deviance was used. Inter‐annual and between‐site differences between nest structures were identified using a multinomial Log‐linear model with an analysis of deviance. Post‐hoc Tukey tests were performed to identify differences between specific seasons and sites.

All analyses were carried out using R statistical software (Version 4.1.0) (R Core Team, 2023) and GraphPad Prism (Version 9.2.0) (GraphPad Software, San Diego, CA, USA). A significance level of *p* < .05 was used.

## 3 RESULTS

### 3.1 Year and site differences in reproductive success

**FIGURE 4:**
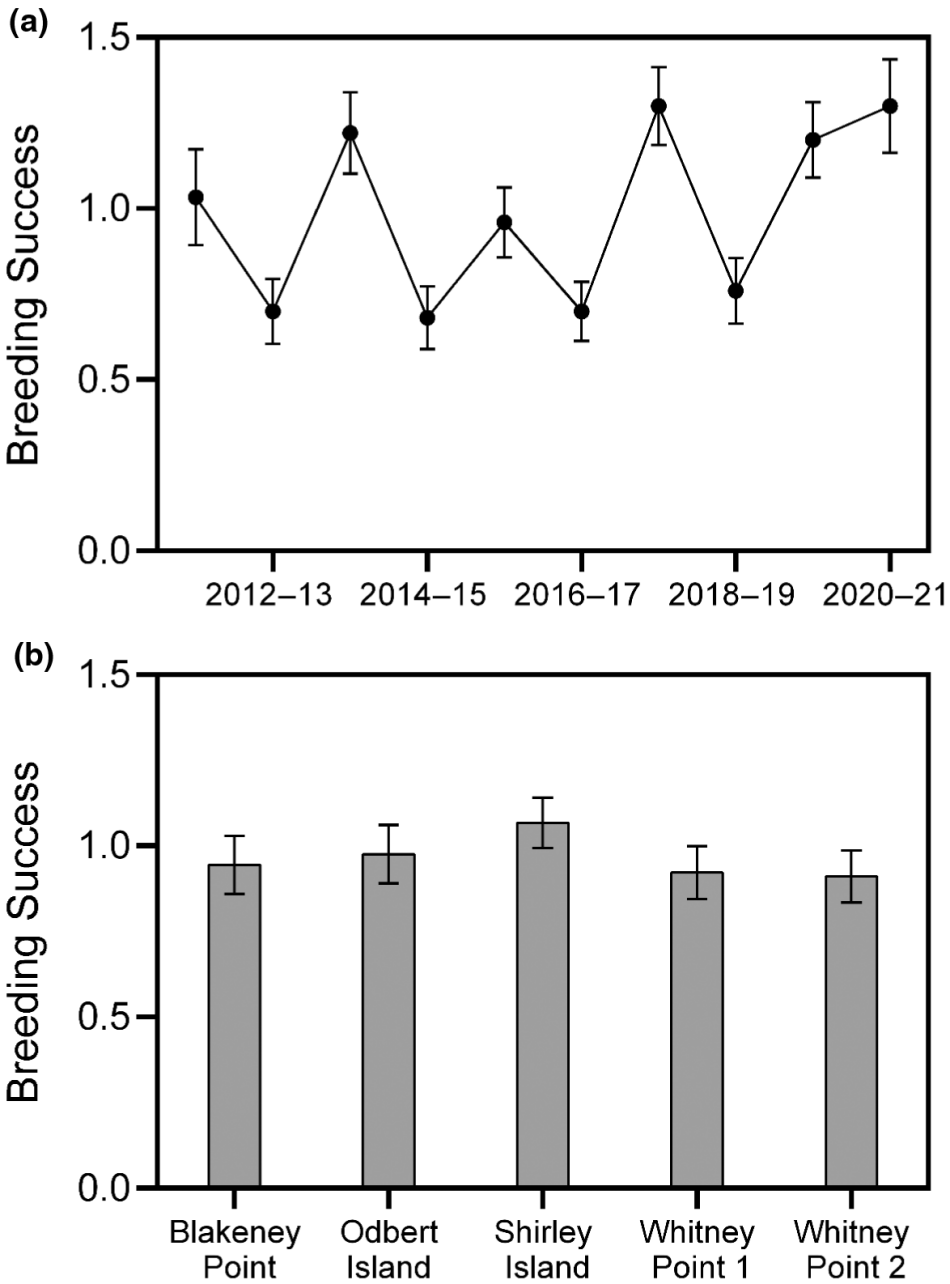
Spatio‐temporal variation in breeding success (chicks crèched per nest) across breeding seasons (a) and camera sites (b). Error bars are standard errors.

From the 450 nests assessed across camera sites and years, there were 313 successes and 137 failures, with 193 single‐chick nests and 120 two‐chick nests. Mean breeding success was 0.96 ± 0.76 SD chicks crèched per nest, with 2017/18 and 2020/21 having the highest yearly breeding success (1.3 chicks per nest), and 2014/15 having the lowest (0.68 chicks per nest; Tukey's post hoc comparisons tests 2017‐18/2014–15: *z* = 2.68, *p* > .05; 2020–21/2014–15: *z* = 2.27, *p* > .05) (Figure 4a). Nest failures most commonly occurred during the guard period, with 54, 80 and 3 failures occurring in incubation, guard and crèche respectively. The 2012/13 and 2014/15 seasons had the highest proportion of failures while 2017/18 had the lowest. Nests at the Shirley Island camera site had the highest average breeding success (1.07 ± 0.69 SD) while nests at the Whitney Point 2 camera site had the lowest (0.91 ± 0.76; Tukey's post hoc comparisons tests *z* = −1.96, *p* > .05) (Figure 4b). There was a significant difference in inter‐annual (likelihood ratio statistic: *L* = 45.9, *p* = <.001, df = 12), and between‐site (likelihood ratio statistic = 7.5, *p* = <.001, df = 8) breeding success as well as a significant year by site interaction (likelihood ratio statistic: *L* = 69.4, *p* = .023, df = 48) (Figure 4).

### 3.2 Drivers of reproductive success

The marine and climatic Cox PH sub‐model did not identify any covariates as having a significant effect on nest survival (Appendix S1). In contrast, significant covariates were identified in the terrestrial and behavioural sub‐models. From the terrestrial sub‐model, the propensity of both moisture (*p* = .018) and snow cover (*p* < .001) were identified as potential drivers of nest survival. In the behavioural sub‐model, an earlier NOD (*p* < .001), nest location away from the colony periphery (*p* = .003), and a high nest structure score (*p* < .001) were all significant factors, with the importance of the latter two varying between breeding stages.

**FIGURE 5:**
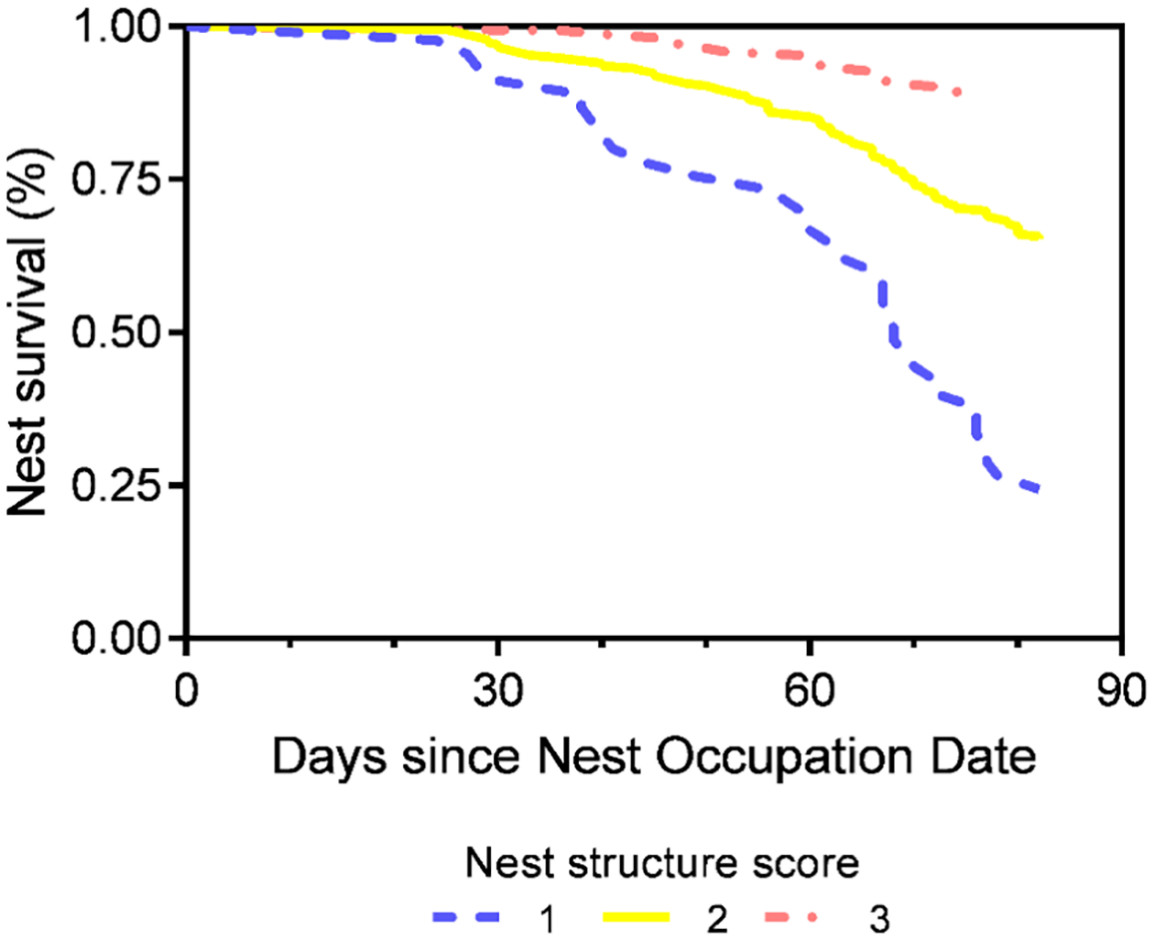
Cox proportional hazards model results showing the proportion of nest survivorship with a nest structure score of 1, 2 and 3.

Based on these results, the final commensurate model identified a mixture of nest characteristics and behavioural covariates as important. During incubation, nests further in the colony (*p* = .022), bigger nests (*p* < .001) (Figure 5) and those that had a lower propensity of snow cover (*p* < .001) all had higher nest survival (Table 1). A nest structure score of either 2 (*p* < .001) or 3 (*p* < .001) (Figure 5) and a lower propensity for the nest to be moist (*p* = .016) had higher survival during the guard period (Table 1). Nest survival was also higher when NOD was earlier (*p* < .001), and lower when there was a higher propensity to accumulate snow cover throughout the breeding season (*p* = .021).

**TABLE 1:** Results of commensurate Cox's proportional hazards model for Adélie penguin nest survival data collected from 2011/12 to 2020/21 at five camera sites within the Windmill Islands region.

Covariate	Hazard ratio	Wald *z*‐score	*p*	Effect on nest survival
Later Nest occupation date	1.10	4.96	<.001	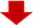
Higher propensity for snow cover	2.58	2.31	.021	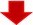
Incubation:Central nest location	0.083	−2.28	.022	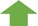
Incubation:Nest structure score 3	0.018	−3.62	<.001	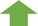
Incubation:Higher propensity for snow cover	5.66	4.02	<.001	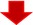
Guard:Nest structure score 2	0.029	−4.54	<.001	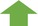
Guard:Nest structure score 3	0.012	−5.71	<.001	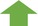
Guard: Higher propensity for nest moisture	4.23	2.41	.016	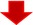

*Note*: The model was highly significant based on the likelihood ratio test (*p* < .001), Wald test (*p* < .001) and score test (*p* < .001). A significance level of *p* < .05 was used. Green arrows represent an increase in nest survival, red arrows represent a decrease in nest survival.

**FIGURE 6:**
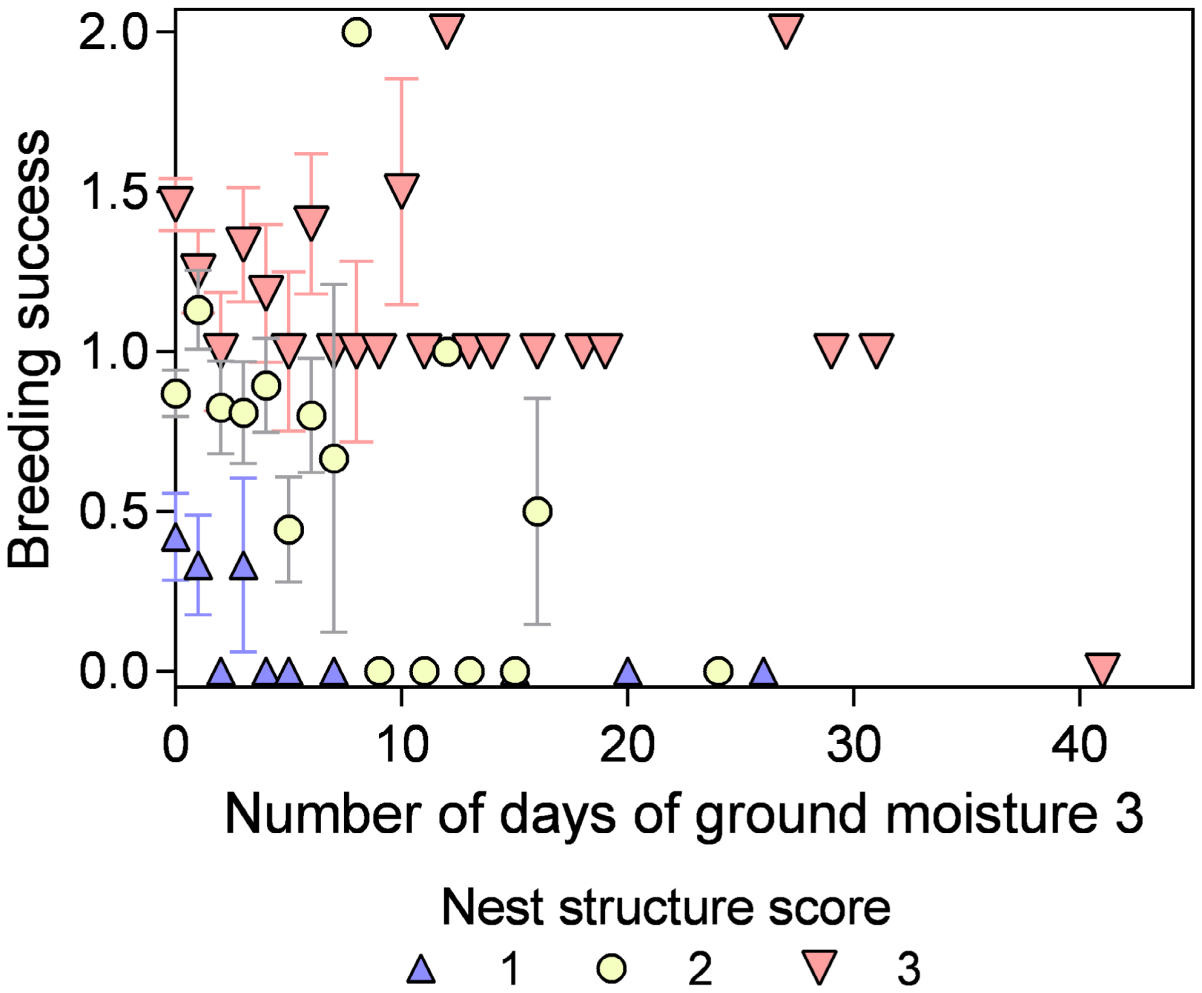
Mean breeding success (±SE) across nests in relation to nest structure score and the number of heavy ground moisture days specifically across all years and sites.

The capacity of nests to allow successful breeding attempts varied according to nest structure scores and in relation to nest moisture (Figure 6). Nests that were smaller and less well constructed were more likely to fail even with less than 5 days of heavy moisture, whereas those with a nest structure score of 3 were able to succeed even with up to 30 days of heavy ground moisture. Nests with structure score 2 were able to withstand more days with heavy ground moisture (up to 9 days) and still successfully rear a chick compared to nests with structure scores of 1, but less than the most well‐constructed nests.

### 3.3 Relationships between covariates

**FIGURE 7:**
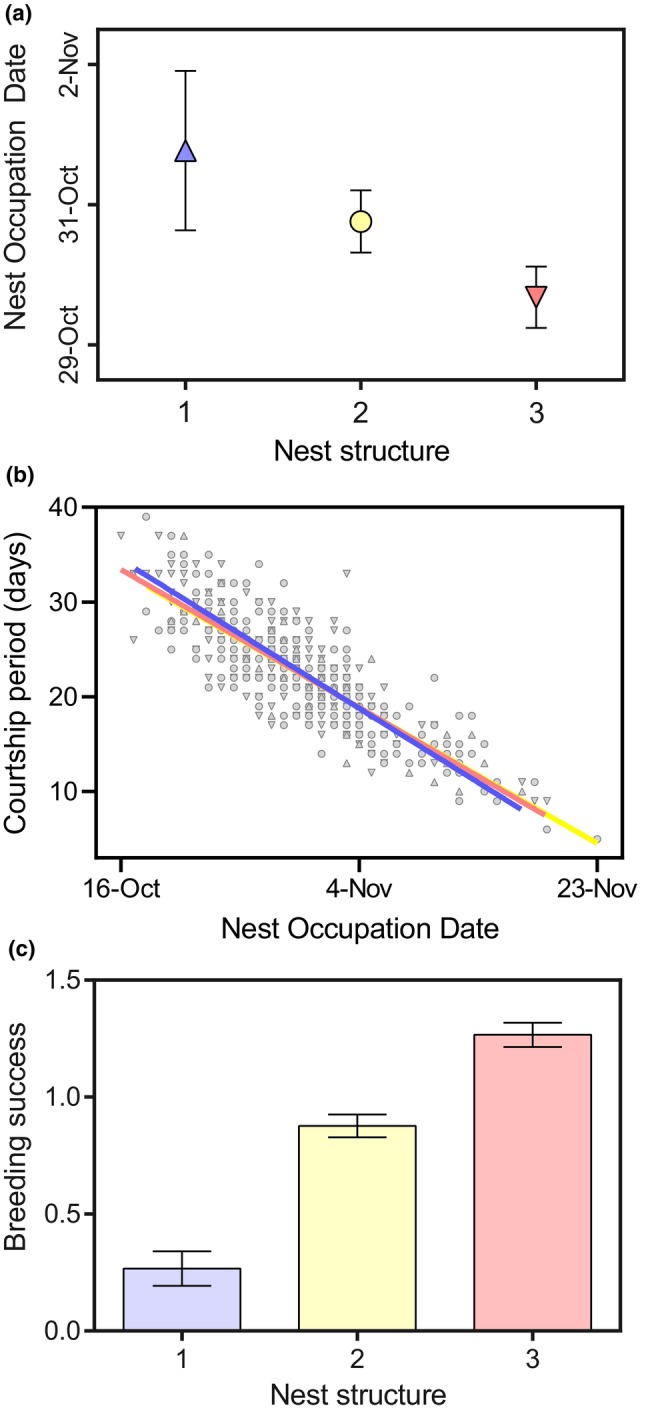
Relationship between (a) Nest structure on December 1 and nest occupation date (NOD), where NOD is a mean (±SE) per each nest structure score; (b) Linear regressions between nest occupation date and courtship period for each nest structure category (derived from FFT—NOD); (c) Mean breeding success (±SE) for each nest structure category on December 1. All means are pooled across years and sites.

Nest structure on December 1 was significantly higher for the nests with an earlier NOD (linear regression: *R* ^2^ = .01, *F =* 4.75, *p* = .02, *n* = 450) (Figure 7a). The time between NOD and FFT date (the courtship period), had a significantly negative relationship with NOD (linear regression: *R* ^2^ = .71, *F =* 1056, *p* = <.001, *n* = 450) (Figure 7b). Finally, nest structure had a significant positive relationship with multinomial breeding success (linear regression: *R* ^2^ = .15, *F =* 78.01, *p* = <.001, *n* = 450) (Figure 7c). No significant relationship was found between nest structure and location on 1 December (linear regression: *R* ^2^ = .008, *F* = 3.629, *p* = .057).

There were statistically significant inter‐annual differences for FFT date (one‐way ANOVA: *F* = 21.6, *p* < .001, df = 6) and NOD (one‐way ANOVA: *F* = 41.65, *p* < .001, df = 6). Similarly, there were significant inter‐annual differences between nest structures (likelihood ratio statistic: *L* = 30.2, *p* < .001, df = 12) and between‐site differences for both nest structure (likelihood ratio statistic = 66.7, *p* < .001, df = 12) and location (one‐way ANOVA: *F* = 9.9, *p* < .001, df = 4).

We identified significant inter‐annual (likelihood ratio statistic: *L* = 130.3, *p* = .004, df = 6) and between‐site differences (likelihood ratio statistic: *L* = 144.46, *p* < .001, df = 4) for number of days with ground moisture score 3 during incubation, and a Tukey's post‐hoc comparisons test showed significant differences between 2018/19 and both 2014/15 (*z* = 3.1, *p* = .03) and 2015/16 (*z* = 3.3, *p* = .01). Blakeney Point had significant differences between Whitney Point 1 (*z* = 4.1, *p* < .001) and Whitney Point 2 (*z* = 3.5, *p* = .004). During guard, there were significant inter‐annual (likelihood ratio statistic: *L* = 229.8, *p* < .001, df = 6) and between‐site differences (likelihood ratio: *L* = 34.6, *p* = .002, df = 4), and 2013/14 and 2015/16 were significantly different to multiple years (Tukey's post‐hoc comparisons test *p* < .05). The crèche period also had significant inter‐annual (likelihood ratio statistic: *L* = 228.8, *p* < .001, df = 6) and between‐site differences between number of days with a ground moisture score 3 (likelihood ratio statistic: *L* = 34.7, *p* = .002, df = 4).

## 4 DISCUSSION

The successful outcomes of seabird reproduction are the cumulative result of environmental (extrinsic) and behavioural (intrinsic) factors which enable seabirds to accommodate a broad range of conditions. While the reproductive success of other populations in East Antarctica are considered to be driven by environmental variables like fast ice (Ballerini et al., 2009; Barreau et al., 2019; Emmerson & Southwell, 2008), in the Windmill Islands it appears to be largely influenced by parental behaviour and how they respond to the environment. This is not unexpected in relation to fast ice, which is less extensive in this location compared to elsewhere. At Béchervaise Island where the fast ice is more extensive, penguin reproductive success is reduced (Emmerson & Southwell, 2008) while areas with less sea ice during the breeding season have higher foraging performance and breeding success (Watanabe et al., 2020). Phenology is also influenced by extensive fast ice delaying nest arrival and clutch initiation (Cimino et al., 2023; Emmerson et al., 2011), as well as other environmental variables like early snowmelt correlating to an earlier clutch initiation (Lynch et al., 2009, 2012). Near Palmer Station there is a negative relationship between late clutch initiation and breeding success, where sea ice and effects from precipitation and island geomorphology delayed clutch initiation (Cimino et al., 2019). Similarly, in our study, we found that a late start of nest occupation influenced the reproductive success of penguins. By examining a suite of marine, climatic, terrestrial and behavioural conditions, we demonstrate the importance of Adélie penguin behaviour in relation to their nest building and maintenance for reproductive success, especially when there is high ground moisture or snow cover in the immediate environment. The presence of moisture and snow cover is a direct product of weather events happening over time, but the duration and intensity of moisture and snow accumulation from such events can be mediated spatially by variations in geomorphology (Cimino et al., 2019). In our study, even though the micro‐habitats and geomorphologies of the five camera sites were visually quite similar, there were differences in ground moisture at the spatial scale of camera sites and these differences were pronounced at the nest scale.

### 4.1 Nest occupation

Our results suggest that an earlier NOD, resulting in earlier clutch initiation, is associated with an increased chance of building a high‐quality nest, and in turn, this strongly benefits reproductive success. It is possible here that, if warmer temperatures in East Antarctica result in increased snowfall early in the breeding season, NOD may be delayed resulting in lower‐quality nests with reduced reproductive success. Alternately, a delay in nest initiation may result in delays in the entire breeding cycle (Barbraud & Weimerskirch, 2006; Emmerson et al., 2011) and the possibility of a mismatch between peak prey consumption requirements and availability of prey (Forcada & Trathan, 2009; Parmesan, 2006; Southwell et al., 2015; Visser & Both, 2005). What is not clear at this stage however, are the more distal factors that determine NOD, which was not only highly influential on reproductive outcomes but also sets the scene for the remainder of the breeding season and is likely to reflect the effect of marine conditions (including prey availability) on the penguin's capacity to prepare for the breeding season or parental experience and quality (Smiley & Emmerson, 2016). Over the 10 years assessed in this study, there were no occurrences of total breeding failure even though there was variability between years, sites and nests. This finding is in contrast to results reported from Béchervaise Island (Emmerson & Southwell, 2008; Irvine et al., 2000) and Pétrels Islands (Ropert‐Coudert et al., 2015) for example, where Adélie penguins can have total or near total breeding failures due to reduced prey, extensive sea ice or precipitation.

### 4.2 Parental investment

Parental investment theory predicts that animals adjust their parental investment to increase offspring survival, with a likely increase in parental investment as the breeding season progresses (Dawkins & Carlisle, 1976; Svagelj et al., 2012; Trivers, 1972). While it is not clear what role parental investment in nest building and maintenance has in relation specifically to the defence of chicks against predators for the Adélie penguin, we would expect penguin parents to modify their efforts in relation to the specific requirements and chick vulnerabilities throughout the breeding season given the challenges of breeding in Antarctica during the short austral summer. Our results suggest that larger nests were advantageous for successful breeding, particularly when the ground was moist as a consequence of snow melt, and that these nests are more likely to be built by pairs that occupy nests early. Consistent with studies on the kelp gull (*Larus dominicanus*), factors such as laying date and parental quality play a key role in nesting attempt outcomes (Svagelj et al., 2012). Our results indicate a benefit under current conditions for individuals who build and maintain well‐constructed nests. In areas like the Windmill Islands and other locations where increased snowfall is expected in the future, building high‐quality nests is advantageous to penguins (Ainley et al., 2010).

It is not unusual for seabirds to derive benefits from nest sites of higher quality in a range of environments (Kokko et al., 2004). Typically, nest quality and breeding success are higher in the centre of the colony (Barbosa et al., 1997; Davis & Mccaffrey, 1986; Ferrer et al., 2014; Mínguez et al., 2001; Morandini et al., 2021). In contrast to other Adélie penguin studies, we did not find a relationship between nest location and structure. This finding is worth further research, as with a growing population and expansion of colonies, the terrestrial habitat and breeding resources may become limiting. Instead, we found that well‐structured nests (i.e., a nest bowl raised off the ground and made with more stones) are more likely to have a higher reproductive success compared to poorly structured nests during incubation and guard periods. It is difficult to discern whether a longer courtship period enables early occupying individuals to put more effort into nest building, or if early arrival and quality nest‐building are complementary behavioural traits (i.e., those birds that arrive early also build and maintain better quality nests). Our results show that nest structure is most important when the egg and chick are highly vulnerable early in the breeding season (Davis, 1982), but of lower importance in mid‐January just before crèche. This is consistent with expectations that chicks require less protection when they are larger and more resilient against detrimental weather conditions (Wilson, 2009) and skua predation (Davis & Mccaffrey, 1986) and can be left for longer between meals (Clarke et al., 2006).

Alongside a well‐structured nest, the deeper a nest was located within the penguin breeding sub colony also positively influenced nest survival. Previous studies show that experienced penguins prefer a central nest location rather than either the very edge or centre of the colony (Ainley, 2002; Ainley et al., 1983; Morandini et al., 2021). At Cape Bird, eggs and chicks at peripheral nests were approximately 20% more susceptible to skua predation compared to those in central nests (Davis & Mccaffrey, 1986). Based on previous observations of isolated nests being unlikely to survive (L. Emmerson, personal observation), we would expect skua predation across the Windmill Islands to render peripheral nest chicks more vulnerable than centrally located chicks. Chinstrap penguins nesting on the periphery are up to eight times more likely to be disturbed than central nesters (Ferrer et al., 2014), and Adélie penguin peripheral nests were more often ‘source’ nests for rock‐stealing (Morandini et al., 2021). However, penguins nesting closer to the periphery rather than deep in the colony may benefit from passing fewer nests on their way to or from their nest, which would reduce the energy needed to defend themselves against attacks from others along the way. Thus, there is a likely trade‐off between having a large nest near the periphery and having to defend it from other individuals who utilise it as a ‘source’ nest for nesting material.

### 4.3 Interactive effects between the environment and nests

We propose that larger nests offer an advantage against undesirable weather conditions including moisture after snow melt by raising the nest bowl away from the ground (Ainley et al., 1983; Fargallo et al., 2001; Moczydłowski, 1989; Stokes & Boersma, 1998). Although nest flooding occurs across a range of ecosystems (Scarton & Valle, 2020; Windhoffer et al., 2017), for polar species, the occurrence of precipitation in the form of rain is uncommon (Robinson et al., 2020) outside of the Western Antarctic peninsula (Chapman et al., 2011; Thompson et al., 1994; Turner et al., 2005), but can occur from water run‐off after snow melt. Historically, precipitation is thought to be responsible for major seabird population changes at other sites in Antarctica (Gao et al., 2018) and is known to affect the breeding success and phenology of Adélie penguins (Boersma, 2008; Hinke et al., 2012). For chinstrap penguins, nests at risk of flooding are thought to have a greater likelihood of failure (e.g., flooding caused a loss of 14% of hatchlings and eggs [Moreno et al., 1995]). Similar to our results, larger nests at some sites have a lower chance of being flooded from meltwater runoff (Moreno et al., 1995; Tenaza, 1971). Our results suggest that although Adélie penguins breeding across the Windmill Islands can cope with small amounts of moisture with low‐quality nests, a higher‐quality nest becomes increasingly important for chick survival when ground moisture levels are high. Although chick survival can be reduced when only 10% of a chicks body is wetted (Chapman et al., 2011), a well‐built nest could be expected to reduce negative impacts from moisture while the chick or egg is kept off the ground within the nest bowl. Nest size may positively reflect parental experience and quality (Fargallo et al., 2001; Moreno et al., 1999), as more effort is required to collect stones for a larger nest, even though this effort can have a clear benefit for reproductive success. Under an experimental setting, chinstrap penguins increased stone provisioning as a response to a reduction of the number of stones making up the nest and heavy snowfall in their surrounds (Fargallo et al., 2001).

In the context of our results indicating negative impacts from high ground moisture on reproductive success, predictions of increasing snowfall as temperatures warm (Ainley et al., 2010) are likely to be detrimental to Adélie penguin reproductive success in the Windmill Islands in the future. An increase in snowfall can reduce the penguins' ability to find their previous nests or the stones required to build them due to a thick snow cover, and this in turn can delay the start of the breeding season and clutch initiation (Lynch et al., 2012). As well, increased snowfall throughout the breeding season can increase mortality and offspring loss, which occurred between 2001 and 2007 in the Ross Sea region (Ainley et al., 2010).

### 4.4 Nesting limitations

Given the benefit of building large nests for nest survival, our results raise a key question: why don't all individuals build large nests with many rocks, independent of their location within the colony? We propose that a penguin pair may fail to build a high‐quality nest for a variety of reasons. Firstly, gathering stones comes with risk from navigating through crowded colonies, and the subsequent attacks and disputes with other individuals (Moreno et al., 1995). Penguins may therefore take the other side of the trade‐off and settle for a nest of lower quality, and expend less energy defending it and reducing the likelihood of becoming a ‘source’ nest. Secondly, the size of the colony relative to nest building material present may influence the resources available for quality nest building and increase competition for stones and territories. This has been demonstrated for chinstrap penguins, where nests in smaller colonies were more likely to have higher quality nests compared to larger sub‐colonies because of competitive pressures for nesting material (Carrascal et al., 1995). If nest material becomes more limited compared to past years, this may be particularly relevant for the Windmill Islands where the Adélie penguin breeding population has increased by six times in the last six decades (Southwell et al., 2021). Thirdly, the structure of a nest may be influenced by an individual bird's experience or age representing an individual's overall quality. Older or more experienced Adélie penguins are more likely to have an earlier clutch initiation date (Polito et al., 2010), which may have contributed to our result of larger, earlier established nests having higher reproductive success. Without additional data, we are unable to determine whether this is due to early arrivals being inclined to build nests of high quality, if early arriving birds have access to a greater abundance of nesting materials or whether those individuals simply had more time to build nests prior to egg lay.

### 4.5 Future work and conclusion

Our study has highlighted the proximal effect of the timing of nest occupation at the beginning of the breeding season on the eventual outcome of reproductive effort at the end of the season and the role of intrinsic factors in this process. We suggest that future studies focus on the more distal, extrinsic features of the marine environment prior to the breeding season that affect the timing of return migration to the breeding colony and subsequent timing of nest occupation. Understanding the interplay between both extrinsic and intrinsic factors in driving populations (de Little et al., 2007; Emmerson & Southwell, 2022; Fay et al., 2015) is crucial for predicting how these populations may respond in the future under a rapidly changing climate.

## AUTHOR CONTRIBUTIONS

**Madi J. McLatchie:** Conceptualization (equal); formal analysis (equal); investigation (lead); methodology (equal); visualization (equal); writing – original draft (lead); writing – review and editing (equal). **Louise Emmerson:** Conceptualization (equal); formal analysis (equal); investigation (supporting); methodology (equal); visualization (equal); writing – original draft (equal); writing – review and editing (equal). **Simon Wotherspoon:** Conceptualization (equal); formal analysis (equal); methodology (equal); visualization (equal); writing – original draft (equal). **Colin Southwell:** Conceptualization (equal); investigation (supporting); methodology (equal); visualization (equal); writing – original draft (equal); writing – review and editing (equal).

## FUNDING INFORMATION

This work was funded by the Australian Antarctic Division through AAS projects 4088 and 4518.

## CONFLICT OF INTEREST STATEMENT

The authors declare no conflict of interest.

## Supporting information


Appendix S1

## Data Availability

The data that support the findings of this study are available through the Australian Antarctic Data Centre (https://doi.org/10.26179/wzem‐0b04) data repository and are available on reasonable request.
