# Supplementary material for: Delay in Adélie penguin nest occupation restricts parental investment in nest construction and reduces reproductive output
Source: Ecol Evol. 2024 Mar 11;14(3):e10988. doi: 10.1002/ece3.10988 (PMC10928351; doi:10.1002/ece3.10988)
Supplement: Supplementary file 1 — Appendix S1 [file ECE3-14-e10988-s001.docx]

Appendices

Appendix S1: Results from marine and climatic, terrestrial, and behavioural Cox proportional hazards survival analysis sub-models. Significant terms are emboldened.

| **Sub-model** | **Covariate** | **P-value** |
| --- | --- | --- |
| **Marine and climatic** |  |  |
|  | Potential foraging overlap | 0.37 |
|  | SOI July-June | 0.099 |
|  | SOI Oct-Jan | 0.39 |
|  | SAM July-June | 0.74 |
|  | SAM Oct-Jan | 0.90 |
|  | Stage:Fast ice | 0.43 |
| **Terrestrial** |  |  |
|  | Windchill | 0.62 |
|  | Days with nest moisture score | 0.14 |
|  | Days with nest snow score | 0.081 |
|  | **Stage:Propensity for nest moisture** | **0.018** |
|  | **Stage:Propensity for snow cover** | **< 0.001** |
| **Behavioural** |  |  |
|  | **Nest occupation date** | **< 0.001** |
|  | First foraging trip | 0.34 |
|  | **Stage: Nest location** | **< 0.001** |
|  | **Stage: Nest structure** | **< 0.001** |

Appendix S2: Years when each camera was operational during this study where grey cells represent periods of operation.

| **Year** | **Blakeney Point** | **Odbert Island** | **Shirley Island** | **Whitney Point 1** | **Whitney Point 2** |
| --- | --- | --- | --- | --- | --- |
| 2011-12 |  |  |  |  |  |
| 2012-13 |  |  |  |  |  |
| 2013-14 |  |  |  |  |  |
| 2014-15 |  |  |  |  |  |
| 2015-16 |  |  |  |  |  |
| 2016-17 |  |  |  |  |  |
| 2017-18 |  |  |  |  |  |
| 2018-19 |  |  |  |  |  |
| 2019-20 |  |  |  |  |  |
| 2020-21 |  |  |  |  |  |
